# Prevention strategies in clinical high-risks states for psychotic disorders: weighing up costs and benefits

**DOI:** 10.1192/j.eurpsy.2023.118

**Published:** 2023-07-19

**Authors:** F. Schultze-Lutter

**Affiliations:** Department of Psychiatry and Psychotherapy, Heinrich-Heine University, Düsseldorf, Germany

## Abstract

**Abstract:**

Today, the indicated prevention of psychosis prior to its first episode is mainly based on clinical high-risk of psychosis (CHR) criteria, namely ultra-high risk criteria and basic symptom criteria. These are associated with conversion-to-psychosis rates of about 30% within three years. Thus, many patients meeting CHR criteria will not progress to psychosis over a medium-term period, and the cost-benefit evaluation of CHR states is always complicated by the largely unknown individual psychosis risk of CHR patients. In consequence, for the lesser risk of adverse events, treatment recommendations commonly favour non-pharmacological strategies, in particular cognitive-behavioural psychotherapy. Yet, individual risk estimation in identified CHR patients is increasingly done with help of machine learning algorithms, which might help to identify CHR patients who would greatly benefit from an additional pharmacological intervention with low-dose antipsychotics. The presentation will discuss the evidence-base of such a multistep, machine learning informed prevention strategy.

**Disclosure of Interest:**

None Declared

